# Correction: Understanding liquid–liquid equilibria in binary mixtures of hydrocarbons with a thermally robust perarylphosphonium-based ionic liquid

**DOI:** 10.1039/d3ra90062b

**Published:** 2023-07-11

**Authors:** Santosh R. P. Bandlamudi, Jimmie L. McGehee, Albaraa D. Mando, Mohammad Soltani, C. Heath Turner, James H. Davis, Kevin N. West, Brooks D. Rabideau

**Affiliations:** a Department of Chemical & Biomolecular Engineering, The University of South Alabama Mobile Alabama 36688 USA brabideau@southalabama.edu +1 251 461-1485 +1 251 460-7147; b Department of Chemistry, The University of South Alabama Mobile Alabama 36688 USA; c Department of Chemical & Biological Engineering, The University Alabama Tuscaloosa Alabama 35487 USA

## Abstract

Correction for ‘Understanding liquid–liquid equilibria in binary mixtures of hydrocarbons with a thermally robust perarylphosphonium-based ionic liquid’ by Santosh R. P. Bandlamudi *et al.*, *RSC Adv.*, 2021, **11**, 31328–31338, https://doi.org/10.1039/D1RA06268A

The authors regret that the funding information was incorrectly shown in the Acknowledgements section of the original manuscript. The corrected funding acknowledgement is as shown below.

The Royal Society of Chemistry apologises for these errors and any consequent inconvenience to authors and readers.

## Supplementary Material

